# Dialysis physicians’ referral behaviors for hemodialysis patients suspected of having cancer: A vignette-based questionnaire study

**DOI:** 10.1371/journal.pone.0202322

**Published:** 2018-08-15

**Authors:** Shingo Fukuma, Miho Kimachi, Kenji Omae, Yuki Kataoka, Hajime Yamazaki, Manabu Muto, Tadao Akizawa, Motoko Yanagita, Shunichi Fukuhara

**Affiliations:** 1 Human Health Sciences, Graduate School of Medicine, Kyoto University, Kyoto, Kyoto, Japan; 2 Department of Healthcare Epidemiology, School of Public Health in the Graduate School of Medicine, Kyoto University, Kyoto, Kyoto, Japan; 3 Center for Innovative Research for Communities and Clinical Excellence, Fukushima Medical University, Fukushima, Fukushima, Japan; 4 Department of Respiratory Medicine, Hyogo Prefectural Amagasaki General Medical Center, Amagasaki, Hyogo, Japan; 5 Department of Therapeutic Oncology, Graduate School of Medicine, Kyoto University, Kyoto, Kyoto, Japan; 6 Division of Nephrology, Department of Medicine, Showa University School of Medicine, Tokyo, Tokyo, Japan; 7 Department of Nephrology, Graduate School of Medicine, Kyoto University, Kyoto, Kyoto, Japan; Universidade Estadual de Campinas, BRAZIL

## Abstract

**Background:**

Although cancer management in dialysis patients has become a commonly encountered issue, known as “onco-nephrology”, few evidence-based clinical recommendations have been proposed. Here, we examined the variation in referral behaviors adopted by dialysis physicians on encountering dialysis patients with signs/symptoms suggestive of cancer.

**Methods:**

We conducted a vignette-based study in August 2015. We sent a 14-page questionnaire to 191 dialysis physicians, including the representative dialysis facilities participating in a Japanese dialysis cohort (the Japan Dialysis Outcomes and Practice Patterns Study). Using vignette scenarios for respiratory, digestive, and urological areas, we assessed the referral behaviors (expert referral or not) adopted by dialysis physicians on encountering dialysis patients with symptoms suggestive of cancer. Each scenario contained three patient functional factors: age (60 or 75 years), performance status (PS 0 or 1), and cognitive dysfunction (absence or presence). We examined the association between physician factors, patient factors, and referral behaviors.

**Results:**

We obtained 94 replies (response rate: 49.2%). For the respiratory scenarios, 38.3% and 51.9% of physicians reported watchful waiting when encountering bilateral and unilateral pleural effusion, respectively. In digestive and urologic scenarios, most physicians (>85%) selected expert referral. We detected differences in referral behaviors between scenarios with different cancer biological factors. However, we found consistency in referral behaviors within the same scenario, even with different patient functional factors (intra-class correlation coefficients within each scenario all >0.7).

**Conclusions:**

Physicians’ referral behaviors for dialysis patients suspected of having cancer vary for different cancer biological factors (probability of having cancer). However, the referral behaviors are similar for different patient functional factors (age, PS, and cognitive dysfunction).

## Introduction

Aging among end-stage renal diseases (ESRD) patients is dramatically advancing worldwide [1], thereby leading to an increased incidence of cancer in those patients [2–5]. However, evidence supporting the efficacy of cancer management regimens in ESRD patients is still lacking [6,7]. Even oncology experts argue as to whether or not aggressive and life-prolonging treatments, such as anticancer agents or radiation, should be administered to ESRD patients, given the lack of evidence supporting the safety of such treatments in this particular population. Further, there is no evidence-based recommendation for patient management after obtained positive results on screening tests related to cancer [8–10]. This situation has resulted in dramatic variation in cancer-related practice patterns for ESRD patients based on the preference of the attending dialysis physician.

Nevertheless, cancers are more likely to be detected in ESRD patients than in the general population, for two possible reasons. First, ESRD patients are more likely to receive cancer screening tests because they regularly visit medical facilities for renal replacement therapy [10–14]. Second, the incidence risk of cancer in ESRD patients may be higher than that in the general population [15,16].

Although cancer management has become a commonly encountered issue with aging of the ESRD population, known as “onco-nephrology”, few evidence-based clinical recommendations have been proposed for the management of such patients [17]. To improve the quality of cancer care, we must first understand the variations in how dialysis physicians decide to refer patients to an oncologist when they observe signs/symptoms suggestive of cancer. Here, we examined the variation in referral behaviors of dialysis physicians by a vignette-based questionnaire study.

## Materials and methods

### Study setting and design

We sent a 14-page questionnaire to physicians at hemodialysis (HD) facilities, including facilities that participated in the Japan Dialysis Outcomes and Practice Patterns Study (J-DOPPS) [18, 19] by mail on August 2015. Respondents to the questionnaires in our study were set as physicians engaged in the dialysis facilities at least once a week and who had worked as a physician for at least five years. In Japan, physicians responsible for dialysis facilities are specialized in nephrology and dialysis therapy and help manage the various health problems faced by dialysis patients, working with patients during their sessions and formulating treatment plans in response to any complaints they may have. Physicians responded to the questionnaires via mail or the web within two months from receipt of the survey booklet. We informed physicians about the aim of this study using a cover letter in the introduction section of the survey booklet. We provided a financial incentive (one thousand yen) to the subjects to encourage their participation. Our present study complied with the Declaration of Helsinki. We did not collect any personal identifying information from the survey participants. This survey was conducted in academic settings with IRB exemption.

### Main outcome

The main outcome was the behavior with respect to consulting oncology experts when they encounter positive signs suggestive of cancer. We inquired about each physician’s referral behavior, asking if they referred the patient to experts (oncologists) promptly or after performing additional examinations, or if they selected watchful waiting instead. We defined those behaviors based on interviews with external experts to cover common referral behaviors in Japan. We included both “prompt referral” and “referral after additional examinations”, as dialysis physicians tend to refer a patient to an expert either after a cancer diagnosis with additional examinations or promptly.

### Characteristics of physicians and facilities

Using a self-reported questionnaire, we evaluated the characteristics of physicians and their facilities. Questionnaires related to physicians inquired about the number of years of physician experience, major specialty, and whether or not they generally perform examinations for patients suspected of having cancer. Questionnaires related to facilities inquired about number of hospital beds, number of hemodialysis beds, type of hospital, distance to the nearest cooperative cancer institution, and number of cancer patients detected in the HD facilities within the past year.

### Vignette scenarios

We assessed the association between patient or physician characteristics and referral behaviors adopted on encountering patients with signs/symptoms related to cancer using a vignette-based study. Vignettes are hypothetical stories describing particular individuals or situations [20–22]. We proposed five vignette scenarios of common clinical situations when physicians might suspect cancer as a differential diagnosis (S1 Appendix): two respiratory scenarios, one digestive scenario, and two urologic scenarios. For each scenario, we varied three patient characteristics, as follows: age (60 or 75 years), performance status (PS 0 [the patient can function in their daily life without difficulty] or 1 [the patient cannot perform strenuous exercises but can perform light work, such house work]), and cognitive dysfunction (absence or presence). Using those three dichotomous factors, eight (2×2×2) patterns were created for each scenario. Specifically, by changing the values of the three factors (age, performance status, and cognitive dysfunction), eight combinations were made (e.g. “60 years old and PS 0 and no cognitive dysfunction”). Respiratory scenario 1 is provided as an example below.

*“Age 75 years*, *male*. *This patient receives maintenance hemodialysis three times a week with a session length of four hours*. *He walks to a hemodialysis center on his own*. *He has cognitive dysfunction* *and* *cannot perform strenuous exercises but can perform light work*, *such as house work [PS 1]*. *One-sided pleural effusion is found on routine chest X-ray before the hemodialysis session at the beginning of the week*. *This examination finding had not been noted two months prior*. *The patient reports feeling fatigue recently but is unaware of any respiratory discomfort.”*

Each physician described their referral behavior for 10 patterns of scenarios (2 randomly selected patterns for each of the 5 scenarios). We developed the vignette scenarios through interviews with four external experts in oncology, general internal medicine, and nephrology; these individuals are mentioned in the Acknowledgements section of this manuscript. We sent drafts of the vignette scenarios to external experts and carefully discussed any content correction. After revising the scenarios according to the experts’ suggestions, all four experts agreed that the vignettes represented common situations in which physicians typically suspect cancer in ESRD patients.

### Statistical analyses

For the characteristics of physicians and their facilities, continuous data with normal distribution were summarized as mean values (standard deviation [SD]), continuous variables with skewed data as median values (inter-quartile range [IQR]), and dichotomous or categorical data as proportions. We assessed the association between patient or physician characteristics and behavior with respect to expert referral after observing signs/symptoms related to cancer. Referral behavior was categorized as a dichotomous variable: “referral (promptly or after additional examinations)” or “watchful waiting”. We calculated the odds ratio (OR) and 95% confidence intervals (CIs), fitting population-averaged panel-data models using generalized estimating equations with a log-link (log-binomial regression model). We adjusted for the patient characteristics of age, PS, and cognitive dysfunction in the vignette scenario in the “minimally adjusted model”. We then added physician characteristics, such as years of physician experience, and facility characteristics, such as type of facilities, distance to the nearest cooperative cancer institution from the HD facility, and number of cancer patients detected at the relevant HD facility within the past year, to the “fully adjusted model”. We calculated intraclass correlation coefficients (ICCs) for each scenario with different patterns of patient characteristics that were randomly selected to examine the consistency of referral behaviors within the same scenario. All statistical analyses were performed using STATA 14.0 (version 14.0; Stata Corp, College Station, TX, USA).

## Results

### Physician characteristics

We sent questionnaire to 191 facilities and obtained 94 replies (response rate: 49.2%). As shown in Table 1, the median number of years of physician experience was 17, and 75% of respondents were nephrologists, and roughly half of facilities were general hospitals. We also examined physicians’ usual methods of cancer management in ESRD patients. A total of 62.8% of physicians reported that they usually refer HD patients suspected of having cancer to an oncologist. Regarding the availability of experts, 43.6% of physicians answered that oncologists were staffed at their facility, and 39.4% answered that oncologists were present within 10 km of their facility. A median of one cancer patients was reported at HD facilities within the past year, and most physicians had no experience with vigorous treatment for cancer, including anticancer agents, radiation, and surgery.

**Table 1:** Baseline characteristics of participants who responded to the questionnaires.

Characteristics	Total (n = 94)	Number of missing data
Years of healthcare experience, years	17 (12–28)	0
Majority, %		0
Nephrologist	75 (79.8)	
Cardiovascular internal medicine	2 (2.1)	
Urology	8 (8.5)	
Surgeon	4 (4.3)	
Pediatrics	0 (0)	
Others	5 (5.3)	
Number of hospital beds, n	302 (19–654)	0
Number of hemodialysis beds, n	26 (15–43)	2
Type of facilities, %		2
General hospital	45 (48.9)	
Hemodialysis facility without inpatient beds	22 (23.9)	
Hemodialysis facility with inpatient beds	25 (27.2)	
Implementation of examination for cancer, %		4
By own facility or department	31 (33.0)	
Commission	59 (62.8)	
Distance to the nearest cooperative institution		9
Within own facility	41 (43.6)	
<1-km radius	7 (7.5)	
1- to <10-km radius	30 (31.9)	
10- to <50-km radius	6 (6.4)	
≥50-km radius	1 (1.1)	
Number of cancer patients in the facilities within the past year, n	1 (0 to 3)	11

Continuous data with a normal distribution were summarized as the mean (standard deviation), continuous variables with skewed data were summarized as the median (interquartile range), and dichotomous or categorical data were summarized as the proportion

### Referral behaviors for respiratory scenarios

We examined the referral behaviors for two respiratory scenarios (S1 Appendix, Scenario 1 and 2). Scenario 1 involved a patient with unilateral pleural effusion, and scenario 2 involved a patient with bilateral pleural effusion. Table 2 shows the referral behaviors for each scenario. The proportion of physicians who selected “referral” was higher in scenario 1 (unilateral pleural effusion) than in scenario 2 (bilateral pleural effusion) (p <0.01). We found that more than one-third of physicians elected to perform additional examinations before expert referral.

**Table 2:** Referral behaviors after observing patient’s symptoms suggestive of cancer using a vignette-based study, n (%).

	Total	
**Respiratory scenario 1 (Scenario 1)**		
Prompt referral to experts	28 (15.3)	113 (61.8)
Referral to experts after additional examinations	85 (46.5)	
Watchful waiting	70 (38.3)	70 (38.3)
**Respiratory scenario 2 (Scenario 2)**		
Prompt referral to experts	26 (14.1)	89 (48.1)
Referral to experts after additional examinations	63 (34.1)	
Watchful waiting	96 (51.9)	96 (51.9)
**Digestive scenario (Scenario 3)**		
Prompt referral to experts	81 (44.0)	182 (98.9)
Referral to experts after additional examinations	101 (54.9)	
Watchful waiting	2 (1.1)	2 (1.1)
**Urological scenario 1 (Scenario 4)**		
Prompt referral to experts	49 (28.5)	164 (95.4)
Referral to experts after additional examinations	115 (66.9)	
Antibiotics and watchful waiting	1 (0.6)	8 (4.7)
Watchful waiting	7 (4.1)	
**Urological scenario 2 (Scenario 5)**		
Prompt referral to experts	40 (23.3)	152 (88.4)
Referral to experts after additional examinations	112 (65.1)	
Antibiotics and watchful waiting	19 (11.1)	20 (11.7)
Watchful waiting	1 (0.6)	

Table 3 shows the association between patient or physician characteristics and referral behaviors. We noted no statistically significant associations between any characteristic and referral behavior in either the minimally or fully adjusted models. The ICCs in scenarios 1 and 2 were 0.91 (0.86 to 0.94) and 0.79 (0.82 to 0.92), respectively (Table 4). Table 5 shows the practice patterns for types of additional examination among physicians who selected “referral to experts after additional examinations”. Most physicians selected CT as the additional examination.

**Table 3:** Association between patient’s and physician’s characteristics and referral behaviors.

	Referral behaviors	
	Respiratory scenario 1	Respiratory scenario 2
**Minimally adjusted model**	**Odds ratio (95% CI)**	
Older patient age	0.99 (0.94 to 1.04)	0.98 (0.92 to 1.04)
Poor PS	0.96 (0.90 to 1.01)	1.04 (0.97 to 1.11)
Presence of dementia	0.96 (0.90 to 1.01)	1.01 (0.94 to 1.08)
**Fully adjusted model**		
Older patient age	0.93 (0.73 to 1.19)	0.88 (0.65 to 1.20)
Poor PS	0.80 (0.61 to 1.05)	1.23 (0.88 to 1.74)
Presence of dementia	0.79 (0.59 to 1.04	1.07 (0.74 to 1.53)
Years of physician experience	1.02 (0.97 to 1.07)	1.00 (0.95 to 1.05)
Type of facility		
General hospital	Reference	Reference
Hemodialysis facility without inpatient beds	3.45 (0.83 to 14.36)	2.98 (0.76 to 11.65)
Hemodialysis facility with inpatient beds	2.30 (0.72 to 7.37)	0.86 (0.29 to 2.51)
Distance to the nearest cooperative cancer institution		
Within own facility	Reference	Reference
<10-km radius	0.40 (0.11 to 1.48)	0.94 (0.28 to 3.09)
≥10-km radius	0.21 (0.032 to 1.42)	0.37 (0.062 to 2.21)
Number of new cancer patients	1.27 (0.95 to 1.70)	1.00 (0.29 to 2.54)

Results shown are the odds ratios (95% confidence intervals), comparing referring the patient to experts promptly or after additional examination with watchful waiting as a reference.

**Table 4:** Consistency of referral behaviors within the same scenario.

	ICC
Respiratory scenario 1 (Scenario 1)	0.91 (0.86 to 0.94)
Respiratory scenario 2 (Scenario 2)	0.79 (0.82 to 0.92)
Digestive scenario (Scenario 3)	0.98 (0.97 to 0.99)
Urological scenario 1 (Scenario 4)	0.73 (0.61 to 0.81)
Urological scenario 2 (Scenario 5)	0.90 (0.85 to 0.93)

Results shown are the intraclass correlation coefficients (ICCs) (95% confidence intervals).

**Table 5:** Additional examination types.

	Prevalence, n (%)
**Respiratory scenario 1 (Scenario 1)**	
Tumor markers	47 (55.3)
Chest CT (simple or contrast-enhanced)	83 (97.7)
Sputum cytology	47 (55.3)
Thoracentesis	38 (44.7)
**Respiratory scenario 2 (Scenario 2)**	
Tumor markers	38 (60.3)
Chest CT (simple or contrast-enhanced)	61 (96.8)
Sputum cytology	38 (60.3)
Thoracentesis	27 (42.9)
**Digestive scenario (Scenario 3)**	
Fecal occult blood retest	68 (68.0)
Tumor markers	35 (35.0)
CT (simple or contrast-enhanced)	33 (33.0)
Abdominal ultrasound	16 (16.0)
Upper gastrointestinal endoscopy	49 (49.0)
Colonoscopy	74 (74.0)
**Urological scenario 1 (Scenario 4)**	
Urinalysis	90 (78.3)
Urinary culture	72 (62.6)
Cytodiagnosis of urine	109 (94.8)
CT (simple or contrast-enhanced)	69 (60.0)
Abdominal ultrasound	58 (50.4)
**Urological scenario 2 (Scenario 5)**	
Urinalysis	90 (80.4)
Urinary culture	81 (72.3)
Cytodiagnosis of urine	94 (83.9)
CT (simple or contrast-enhanced)	64 (57.1)
Abdominal ultrasound	51 (45.5)

### Referral behaviors for digestive scenario

We examined the referral behaviors in one digestive scenario (S1 Appendix, Scenario 3). This scenario involved a patient who was positive for fecal occult blood test (FOBT). Table 2 shows that most physicians selected “referral”. We found that more than half of physicians elected to perform additional examinations before expert referral. We did not examine the association between the patient or physician characteristics and referral behaviors in the digestive scenario, because the number who selected “watchful waiting” was too small to estimate the OR. Table 4 shows that the ICC for scenario 3 was 0.98 (range: 0.97 to 0.99). Table 5 shows that 74% of physicians selected colonoscopy for an additional examination, while 68% selected FOBT, which is not recommended for patients with positive findings on an initial FOBT.

### Referral behaviors for urological scenario

We examined referral behaviors in two urological scenarios (S1 Appendix, Scenario 4 and 5). Scenario 4 involved a patient with repetitive hematuria, and scenario 5 involved a patient with new-onset hematuria accompanied by lower abdominal pain. Table 2 shows that most physicians selected “referral” for both scenarios. We found that about two-third of physicians elected to perform additional examinations before expert referral. We did not examine the association between patient or physician characteristics and referral behaviors for the same reason as with the digestive scenario. Table 4 shows that the ICC was 0.73 (0.61 to 0.81) for scenario 4 and 0.90 (0.85 to 0.93) for scenario 5. Table 5 shows that a greater proportion of physicians selected urinalysis, urinary culture, or cytodiagnosis of urine than CT or abdominal ultrasound.

## Discussion

Referral behaviors adopted when physicians encountered positive results related to respiratory cancer ranged from “referral to experts” to “watchful waiting”. In digestive and urologic scenarios, most physicians selected “referral to experts”. We noted such variation in referral behaviors between different scenarios but consistency within the same clinical scenarios (high ICC). We also found that many physicians elected to perform additional examinations before expert referral. These results suggest that physician’s referral behaviors might be more strongly affected by biological factors (probability of cancer) than patient’s functional factors, such as age, PS, and presence of cognitive dysfunction. This study is the first to clarify physicians’ preferences with respect to referral behaviors for cancer among ESRD patients.

In this study, we noted no significant associations between patient characteristics (age, PS and cognitive function) and referral behaviors, perhaps because we assessed the referral behaviors before a diagnosis rather than the treatment selection after a diagnosis. Even in cases in which physicians assumed a poor prognosis based on the patient characteristics, they may have selected “referral to expert” to confirm the diagnosis and evaluate the prognosis.

No international guidelines or recommendations for cancer management in ESRD patients have yet been established, although cancer has become a common issue encountered in such patients. Several previous studies suggested that ESRD patients may be at an increased risk for certain cancers due to multiple abnormalities, such as immune disorders, genetic disorders, chronic oxidative stress, viral infections, vitamin D deficiency, immuno-suppressive agent use, and inherent renal diseases like acquired cystic kidney disease [23–26]. The results from this study will prove useful for generating discussion regarding cancer management in ESRD patients [4, 8, 27].

We observed variations in referral behaviors for respiratory cancer based on the disease characteristics. In contrast to members of the general population, pleural effusion is occasionally observed in HD patients on regular chest X-ray due to fluid overload [28]. This may explain why nearly 40%-50% of physicians selected “watchful waiting” on encountering a patient with pleural effusion. Of note, the proportion who selected “watchful waiting” was higher in cases of bilateral pleural effusion than in those with unilateral pleural effusion. “Watchful waiting” is clinically appropriate for bilateral pleural effusion, as this finding is strongly suggestive of transudate (i.e. a benign finding) [29]. Among physicians who selected additional examinations, most chose CT, which is consistent with the recommendations outlined in clinical guidelines in situations where thoracentesis is not possible [30]. However, half of respondents also selected sputum cytology, which is not recommended for additional examinations of lung cancer [31]. To assess practice variations, we tried to develop scenarios involving the potential (not definite) presence of cancer. The respiratory scenarios were to represent situations in which physicians might suspect cancer as a differential diagnosis.

In terms of digestive cancer, most physicians (more than 98%) responded that they would “referral to experts” after noting positive signs on FOBT. A recent systematic review (8 studies, n = 38718) described the diagnostic abilities of the FOBT for colorectal cancer; the sensitivities and specificities were 73% and 96% with a single stool specimen [32]. Given the extremely high specificity of the FOBT for colorectal cancer, consulting gastroenterologists for a closer investigation when encountering positive results on this test seems a reasonable course of action. Among physicians who selected additional examinations, three-quarters selected colonoscopy, which is consistent with the recommendations of clinical guidelines [33]. However, 68% of physicians responded that they would repeat the FOBT. A previous study suggested little to no benefit in repeating the FOBT for individuals with positive results on an initial test [34].

For urologic cancer, most physicians responded that they would “referral to experts” after observing hematuria. This finding indicates that most physicians followed the established guidelines recommending urgent urologic evaluation in all adults with gross hematuria [35, 36]. However, 5%-12% of physicians selected “watchful waiting” after observing hematuria. A number of physical conditions unique to dialysis patients, such as oliguria or anuria, low vesical capacity, and atrophic renal transformation with multiple cysts, may have led to these physicians’ negative behavior toward standard examinations of gross hematuria, including urinalysis and abdominal ultrasound. In scenario 5 (urological scenario 2) in particular, a large proportion of physicians tended to be reluctant to perform examinations in a patient with lower abdominal pain in addition to hematuria, which are apparently symptoms of simple cystitis. Interestingly, even among physicians who selected additional examinations, the proportion selecting CT or abdominal ultrasound was relatively low. Difficulty in interpreting results related to a differential diagnosis in dialysis patients, as mentioned above, might have influenced the practice patterns adopted.

Several strengths to the present study warrant mention. First, this study was the first to examine the referral behaviors with ESRD patients suspected of having cancer. Strategies for managing cancer in ESRD patients have recently been discussed under the concept of “onco-nephrology”, although studies in this area are still lacking [17, 37]. This study was the first step towards establishing good collaboration between dialysis physicians and oncologists. Second, we examined variations in practice patterns between different clinical scenarios through a vignette-based study. Such studies are useful for evaluating the preferences, beliefs, and common attitudes of subjects using a hypothetical scenario representing a particular situation where recommendations for examination or treatment have not yet been established, such as with cancer management for ESRD patients. Third, we sent questionnaires to physicians at HD facilities throughout Japan, including facilities that participated in J-DOPPS, a study involving randomly selected representative HD facilities in Japan. However, several limitations to the present study also warrant mention. First, we only examined three types of cancer, and our findings may not be generalizable to other types. However, the types assessed in the present study are common in HD patients [6, 7], and we believe that our findings should prove useful for the evaluation of behaviors in most HD patients. Second, the patient characteristics in the scenarios were limited to three factors: age, PS, and cognitive dysfunction. This is because the pattern of the scenario increases synergistically, as the number of factors in the scenario increases. Therefore, we were unable to assess other factors, such as socio-economic factors. Third, this study included only Japanese physicians. Differences in healthcare systems among countries may affect the generalizability of our results. Fourth, the sample size was rather small, despite our efforts to include as many accessible physicians as possible within our budget constraints. Because of the limited number of participants in this study, the generalizability of our results merits consideration. To assess the representative referral behaviors in a real-world setting, we should include a larger number of randomly-sampled physicians in a future study. Fifth, informing participants about the aim of this study may have affected their answers, although we used vignettes and asked participants to select behaviors that fit their usual practice. Finally, respondents’ stated preferences are not always consistent with their real-world practice. However, the possibility of such a gap between the responses and actual actions is inherent in self-reported questionnaire studies.

## Conclusions

The referral behaviors adopted when dialysis physicians encounter cancer-related positive signs in ESRD patients vary among cancer biological factors. The patient functional factors of age, PS, and cognitive dysfunction may not affect those referral behaviors. To our knowledge this is the first study to examine physicians’ behaviors with respect to referral to experts when encountering ESRD patients suspected of having cancer. Future studies should evaluate the association between these behaviors and the patient’s clinical outcomes and establish an outcome-based management strategy for cancer in ESRD patients.

## Supporting information

S1 Appendix Five vignette scenarios for two lung cancer situations, one digestive cancer situation, and two urological cancer situations. (DOC)

S1 Dataset Dataset of the characteristics of physicians and their facilities along with responses to vignette-based scenarios. (XLSX)
