# Supplementary material for: Dialysis physicians’ referral behaviors for hemodialysis patients suspected of having cancer: A vignette-based questionnaire study
Source: PLoS One. 2018 Aug 15;13(8):e0202322. doi: 10.1371/journal.pone.0202322 (PMC6093689; doi:10.1371/journal.pone.0202322)
Supplement: S1 Appendix — (DOC) [file pone.0202322.s001.doc]

***Respiratory scenario 1 (Scenario 1)***

(1) Age 60 (or 75) years, male. This patient receives maintenance hemodialysis three times per week with a session length of four hours. He walks to a hemodialysis center on his own. He (2) has (or doesn’t have) cognitive dysfunction, and (3) he can live his daily life without difficulty (PS 0) (or he cannot perform strenuous exercises but can perform light work such as house work [PS 1]).

One-sided pleural effusion is found on routine chest X-ray before the hemodialysis session at the beginning of the week. This examination finding had not been noted two months prior. The patient reports feeling fatigue recently but is unaware of any respiratory discomfort.

*Q1. How do you deal with this situation next?*

1. I refer this patient to the Department of Respiratory Medicine.
2. I perform an additional examination, and if any abnormal findings are noted, I refer this patient to the Department of Respiratory Medicine.
3. I decrease the patient’s dry weight and make a follow-up observation.

*Q2. Please answer this question if you responded b) in Q1. Which additional examinations do you order? Please select all of the following alternatives that apply:*

1. Tumor markers
2. CT (simple or contrast-enhanced)
3. Sputum cytology
4. Thoracentesis

***Respiratory scenario 2 (Scenario 2)***

(1) Age 60 (or 75) years, male. This patient receives maintenance hemodialysis three times per week with a session length of four hours. He walks to a hemodialysis center on his own. He (2) has (or doesn’t have) cognitive dysfunction, and (3) he can live his daily life without difficulty (PS 0) (or he cannot perform strenuous exercises but can perform light work such as house work [PS 1]).

The patient recently experienced appetite loss and prolonged coughing. Bilateral pleural effusion is found on routine chest X-ray before the hemodialysis session at the beginning of the week.

*Q1. How do you deal with this situation next?*

1. I refer this patient to the Department of Respiratory Medicine.
2. I perform an additional examination, and if any abnormal findings are noted, I refer this patient to the Department of Respiratory Medicine.
3. I decrease the patient’s dry weight and make a follow-up observation.

*Q2. Please answer this question if you responded b) in Q1. Which additional examinations do you order? Please select all of the following alternatives that apply:*

1. Tumor markers
2. CT (simple or contrast-enhanced)
3. Sputum cytology
4. Thoracentesis

***Digestive scenario (Scenario 3)***

(1) Age 60 (or 75) years, male. He walks to a hemodialysis center on his own. He (2) has (or doesn’t have) cognitive dysfunction, and (3) he can live his daily life without difficulty (PS 0) (or he cannot perform strenuous exercises but can perform light work such as house work [PS 1]).

Occult blood was found to be positive in an annual screening examination.

*Q1. How do you deal with this situation next?*

1. I refer this patient to the Department of Gastrointestinal Medicine.
2. I perform additional examinations (except for occult blood test), and if any abnormal findings are noted, I refer this patient to the Department of Gastrointestinal Medicine.
3. I make a follow-up observation if there are no symptoms.

*Q2. Please answer this question if you responded b) in Q1. Which additional examinations do you order? Please select all of the following alternatives that apply:*

1. Fecal occult blood retest
2. Tumor markers
3. CT (simple or contrast-enhanced)
4. Abdominal ultrasound
5. Upper gastrointestinal endoscopy
6. Colonoscopy

***Urological scenario 1 (Scenario 4)***

(1) Age 60 (or 75) years, male. He walks to a hemodialysis center on his own. He (2) has (or doesn’t have) cognitive dysfunction, and (3) he can live his daily life without difficulty (PS 0) (or he cannot perform strenuous exercises but can perform light work such as house work [PS 1]).

During rounds, the patient says that he noted hematuria three days prior, but the symptom was improved today. He also noted the same symptom three weeks prior, but it also improved after only a few days (Please answer if you are not an expert of urology.).

*Q1. How do you deal with this situation next?*

1. I refer this patient to the Department of Urology.
2. I perform additional examinations, and if any abnormal findings are noted, I refer this patient to the Department of Urology.
3. I administer antibiotics and adopt a wait-and-see approach.
4. I make a follow-up observation because the patient feels that the symptom has improved.

*Q2. Please answer this question if you responded b) in Q1. Which additional examinations do you order? Please select all of the following alternatives that apply:*

1. Urinalysis
2. Urinary culture
3. Cytodiagnosis of urine
4. CT (simple or contrast-enhanced)
5. Abdominal ultrasound

***Urological scenario (Scenario 5)***

(1) Age 60 (or 75) years, male. He walks to a hemodialysis center on his own. He (2) has (or doesn’t have) cognitive dysfunction, and (3) he can live his daily life without difficulty (PS 0) (or he cannot perform strenuous exercises but can perform light work such as house work [PS 1]).

During rounds, the patient says that he felt discomfort of the lower abdomen three days prior and has had hematuria since the previous night. He has no other symptoms, such as a fever.

*Q1. How do you deal with this situation next?*

1. I refer this patient to the Department of Urology.
2. I perform additional examinations, and if any abnormal findings are noted, I refer this patient to the Department of Urology.
3. I administer antibiotics and adopt a wait-and-see approach.
4. I make a follow-up observation because the patient feels that the symptom has improved.

*Q2. Please answer this question if you responded b) in Q1. Which additional examinations do you order? Please select all of the following alternatives that apply:*

1. Urinalysis
2. Urinary culture
3. Cytodiagnosis of urine
4. CT (simple or contrast-enhanced)
5. Abdominal ultrasound
